# Characterizing heterogeneity of non‐small cell lung tumour microenvironment to identify signature prognostic genes

**DOI:** 10.1111/jcmm.16092

**Published:** 2020-11-12

**Authors:** Kai Mi, Fuhui Chen, Zhipeng Qian, Jing Chen, Dongxu Lv, Chunlong Zhang, Yanjun Xu, Hongguang Wang, Yuepeng Zhang, Yanan Jiang, Desi Shang

**Affiliations:** ^1^ College of Bioinformatics Science and Technology Harbin Medical University Harbin China; ^2^ Department of Respiratory The Second Affiliated Hospital of Harbin Medical University Harbin China; ^3^ School of Civil Engineering Northeast Forestry University Harbin China; ^4^ Key Laboratory of Bio‐based Material Science and Technology (Ministry of Education) School of Material Science and Engineering Northeast Forestry University Harbin China; ^5^ Department of Pharmacology (State‐Province Key Laboratories of Biomedicine‐ Pharmaceutics of China, Key Laboratory of Cardiovascular Research, Ministry of Education) College of Pharmacy Harbin Medical University Harbin China; ^6^ Translational Medicine Research and Cooperation Center of Northern China Heilongjiang Academy of Medical Sciences Harbin China

**Keywords:** non‐small cell lung cancer, prognostic, tumour microenvironment

## Abstract

Growing evidence has highlighted the immune response as an important feature of carcinogenesis and therapeutic efficacy in non‐small cell lung cancer (NSCLC). This study focused on the characterization of immune infiltration profiling in patients with NSCLC and its correlation with survival outcome. All TCGA samples were divided into three heterogeneous clusters based on immune cell profiles: cluster 1 ('low infiltration' cluster), cluster 2 ('heterogeneous infiltration' cluster) and cluster 3 ('high infiltration' cluster). The immune cells were responsible for a significantly favourable prognosis for the 'high infiltration' community. Cluster 1 had the lowest cytotoxic activity, tumour‐infiltrating lymphocytes and interferon‐gamma (IFN‐γ), as well as immune checkpoint molecules expressions. In addition, MHC‐I and immune co‐stimulator were also found to have lower cluster 1 expressions, indicating a possible immune escape mechanism. A total of 43 differentially expressed genes (DEGs) that overlapped among the groups were determined based on three clusters. Finally, based on a univariate Cox regression model, prognostic immune‐related genes were identified and combined to construct a risk score model able to predict overall survival (OS) rates in the validation datasets.

## 1 INTRODUCTION

Successful lung cancer management remains an elusive subject in medicine. Besides being the leading cause of worldwide cancer‐related mortality,^1^ non‐small cell lung cancer (NSCLC) is the most common and severe lung malignancy subtype. Histologically, NSCLC exists as either large cell carcinoma, squamous cell carcinoma and adenocarcinoma. Despite the clinical gains of advances in biologically targeted agents and chemotherapy, many patients experience resistance to these modalities, causing poor survival rates for advanced‐stage NSCLC patients.^2^, ^3^ Therefore, more effective prognostic and therapeutic strategies are also urgently needed. Growing research has demonstrated the essential role of the immune system in the development and progression of NSCLC. The immune tumour microenvironment is considered an integral component and hallmark of NSCLC, which consists of multiple immune and stromal cells, as well as some immunomodulators.^4^ In addition, the tumour immune microenvironment of patients with NSCLC can also predict tumour recurrence, overall survival and response/resistance to anti‐cancer therapy.^5^, ^6^ The application of immunotherapy is a milestone in NSCLC treatment. Immune checkpoints are critical modulators of the immune system's function. Immunotherapies targeting various immune checkpoints, including programmed death 1 (PD‐1), programmed death‐ligand 1 (PD‐L1), Cytotoxic T‐Lymphocyte Antigen 4 (CTLA4) and programmed cell death 1 ligand 2 (PD‐L2), have a beneficial impact on NSCLC.^7^, ^8^, ^9^ Besides, as front‐line therapeutic agents, these immune inhibitors have shown promising results compared with the other unsuccessful treatments. Although cancer immunotherapy in the treatment of NSCLC provides a sustained clinical response, some patients receiving immunotherapy still have a poor prognosis and serious adverse reactions.^10^, ^11^ Given that the effectiveness of immunotherapy depends partly on the heterogeneity of the tumour microenvironment,^12^, ^13^ characterizing the heterogeneity of the tumour microenvironment can also help in accurately predicting outcome in NSCLC patients.

A detailed analysis of tumour‐immune interaction in NSCLC is important hence this research examines the relative quantity and prognostic characteristics of immune cell infiltration into NSCLC tissues. Patient studies were divided into three immune clusters based on the outcome of single sample GSEA (ssGSEA), and the relative proportions of different immune cell groups were estimated using the CIBERSORT algorithm. The prognostic value and the possible signatures of immune‐related genes were then characterized.

## 2 MATERIALS AND METHODS

### 2.1 Data sets preprocessing

To collect gene‐expression data from the Cancer Genome Atlas (TCGA), the UCSC Xena browser (GDC hub: https://gdc.xenahubs.net) was used. In total, 1016 individuals with NSCLC (lung adenocarcinoma: 515; lung squamous cell carcinoma: 501) were enrolled in this study. Publicly available lung cancer (NSCLC) gene expression data sets containing comprehensive clinical annotations were extracted from the Gene Expression Omnibus (GEO) dataset. In addition, 5 validation cohorts (GSE11969, n = 163; GSE13213, n = 118; GSE30219, n = 308; GSE41271, n = 275; GSE42127, n = 176) were obtained from the GEO database (https://www.ncbi.nlm.nih.gov/geo/). The clinical features of patients, including age, tumour stage, gender, survival time, histological type and outcome, were also obtained. Patients without clinical evidence were excluded from the study.

### 2.2 Immune‐clusters characterization and cell abundance

The enrichment scores of 782 genes representing 27 immune cell types were determined using the ssGSEA software implemented in the R GSVA package.^14^ Using the CIBERSORT method, relative proportions of infiltrating immune cells were evaluated.^15^ CIBERSORT is based on a linear support vector regression that estimates the degree of immune cell infiltration. ESTIMATE was used to calculate immune and stromal scores. This algorithm can derive composite scores based on the level of immune cell infiltration in tumour tissues and the amount of stromal cells present.^16^ Immune cytotoxic activity (CYT) was determined based on a previously published formula.^17^ The molecules perforin‐1 (PRF1) and granzyme A (GZMA), considered to be closely linked to CD8+ T cell activation,^17^ were used to calculate the CYT.^18^, ^19^ The signature of tumour inflammation (TIS) is an 18‐gene signature designed solely for research and can measure background adaptive immune responses suppressed in tumours. The TIS score is estimated as the average of log2‐FPKM gene expression of the marker genes.^20^, ^21^ The relative antigen presentation machinery (APM) was determined for the major histocompatibility complex (MHC) molecules.^14^ The density of T cell correlates positively with a better prognosis in many human cancers.^22^, ^23^ To investigate tumour‐infiltrating T cells, the proportion of tumour‐infiltrating lymphocytes (TILs) was calculated.^24^, ^25^, ^26^, ^27^

### 2.3 Hierarchical clustering

For Hierarchical agglomerative clustering of NSCLC microenvironment based on ssGSEA result, a total of four immune checkpoints were included namely; CTLA4, PD‐L1, PD‐L2 and PD‐1. These molecules are present on T cell surfaces and are checkpoint receptor inhibitors that would otherwise activate T cells.^28^, ^29^

### 2.4 Differential analysis of expressed genes

DEGs were extracted with the R package limma to identify genes associated with tumour microenvironment infiltration.^30^ Statistical significance was set at (|log FC| > 1, *P* < .01) as previously implemented.

### 2.5 Enrichment analysis

The Kyoto Encyclopedia of Genes and Genomes (KEGG) analysis and the Database for Annotation, Visualization and Integrated Discovery (DAVID) tool were used to further explore DEG functions.

### 2.6 Definition of risk score

To build a predictive model related to the prognosis, DEGs underwent a univariate Cox regression model to assess their strength in predicting survival. Only 2 of 43 genes (ENO1, SLC34A2) showed the ability to evaluate patient survival independently, with both genes having statistically significant levels at .05. A two‐gene risk score (RS) model for OS prediction was developed by combining the expression data from both genes with their corresponding coefficient of univariate cox result. The following formula was used: RS = (0.2771 × expression value of ENO1) + (−0.1551 × expression value of SLC34A2) (Table S2). The RS model was calculated for all individuals in the validation dataset.

### 2.7 Statistical analysis

R software (version 3.5.0) was used to perform all statistical analyses using the Student's *t* test. Pearson's correlation coefficients were used to assess the relationship between continuous variables. To conduct a survival analysis of all three clusters, the Kaplan‐Meier approach was used, with the subsequent findings compared using the log‐rank test. ANOVAs and Tukey's multiple comparison tests (*P‐value < .05; **P‐value < .005; ***P‐value < .0005; ****P‐value < .00005) were used to determine the distribution of inflammation markers and within immune clusters utilizing box plots. Heat maps were generated by the function of pheatmap_1.0.10.

## 3 RESULTS

### 3.1 Landscape of the microenvironment phenotypes

**Figure 1:**
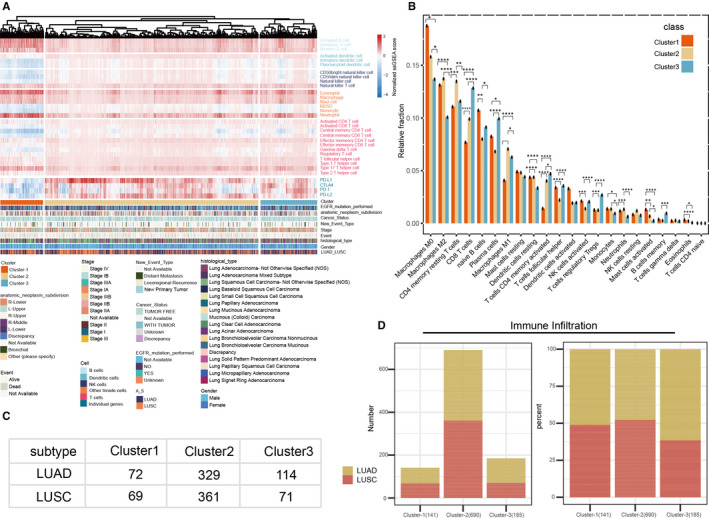
Tumour microenvironment phenotypes. A, Hierarchical agglomerative clustering of 1016 TCGA cohort patients based on ssGSEA scores from 27 types of immune cells. Gender, anatomic primary tumour location, stage, subtype, mutation status of EGFR and survival rates are annotated in the lower panel. Three distinct immune infiltration clusters, termed cluster 1, cluster 2 and cluster 3, are also described. B, Bars represent the mean proportion of 22 populations of immune cells as obtained from the CIBERSORT results. The *x*‐axis represents immune cell population, whereas the y‐axis represents mean proportion of the immune cell component with standard error. C, Immune infiltration based on the outcome of ssGSEA in the immune cluster (D) the composition ratio and absolute values of three immune cluster. *P < .05; **P < .005; ***P < .0005; ****P < .00005

To create a tumour microenvironment profile for NSCLC, 782 genes representing 27 types of immune cells (Table S1) were analysed. For all the 1016 NSCLC samples, the ssGSEA method was used to estimate the richness of each gene. Subsequently, based on hierarchical clustering analysis, immune‐related NSCLC subtypes were determined. All patients were grouped into three clusters, namely; cluster 1, cluster 2 and cluster 3 (Figure 1A). Cluster 3 contained the highest degree of immune cell infiltration in comparison with the other two clusters. CIBERSORT method was used to evaluate the proportions of immune cell populations (Figure 1B). The result was characterized by an abundance of infiltration of macrophages M0, naive B cells, activated dendritic cells. Cluster 3 showed markedly increased levels of CD8 T cell infiltration. Finally, following the tumour phenotype and the cluster, we demonstrated the complete immune cell infiltration profile within NSCLC (Figure 1C,D). In comparison with the other clusters, we noted that the degree of immune infiltration was higher in cluster 2 (LUAD = 329, LUSC = 361). Interestingly, there was also a slight subtype difference in the immune infiltration of cluster 3 (LUAD = 114, LUSC = 71 in cluster 3), which could lead to variations of survival outcome.

### 3.2 Prognostic significance of immune cells

**Figure 2:**
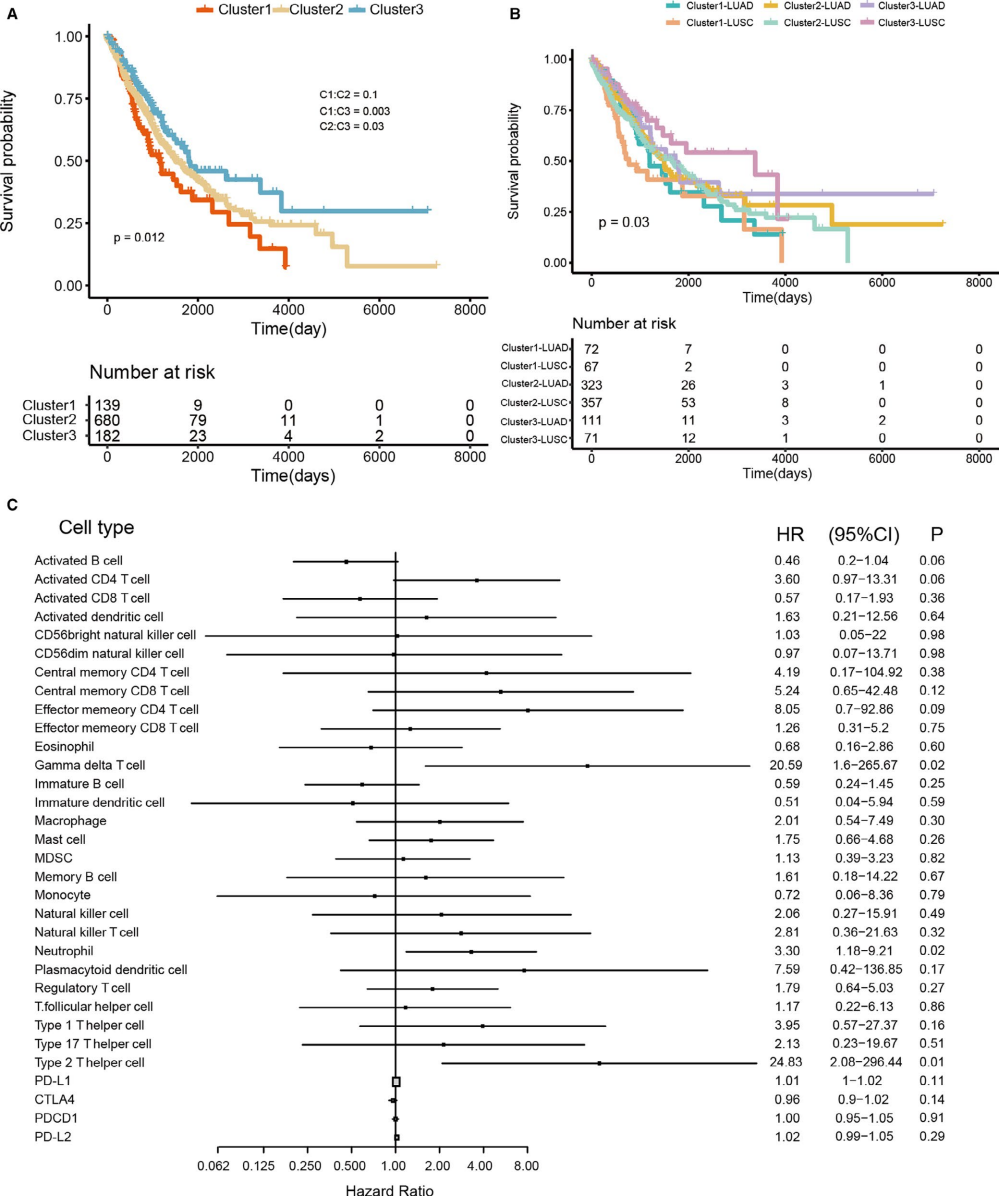
Prognostic significance of immune cells. A, Overall survival (OS) of all NSCLC patients based on tumour microenvironment infiltration classes is shown as Kaplan‐Meier curves, with the Log‐rank test, show overall *P* = .012. B, Overall survival (OS) of all NSCLC patients based on tumour microenvironment infiltration classes by NSCLC subtype are shown as Kaplan‐Meier curves, with the Log‐rank test overall *P* = .03. C, The forest plot for the ssGSEA result. The univariable Cox model was used to determine statistical significance

The clinical association between the immune profiles of all three clusters and the resultant survival of patients was investigated. Compared with other clusters (Figure 2A), we observed that individuals with the cluster 3 type immune infiltration profile had significantly better overall survival (OS; log‐rank *P* = .012). In addition, we divided NSCLC patients into LUAD and LUSC to explore the possible correlation between prognostic success and subtype (LUAD and LUSC). Survival curves are shown in Figure 2B. The most favourable OS was found in the cluster 3‐LUSC and 3‐LUAD cohort, highlighting the role of the cluster 3 subset in providing immunity against tumour activity in NSCLC. In patients with cluster 3 trends (HR = 0.66; 95% CI = 0.46‐0.96; *P* = .03; Figure S1), the multivariate Cox proportional hazard model revealed a greater OS. To evaluate whether 27 human immune cell phenotypes and 4 immune checkpoint molecules could affect the outcome of patients, we performed univariate cox regression (Figure 2C). The overall survival was significantly associated with gamma delta T cells, neutrophils and Type 2 T helper cells (gamma delta T cell: *P* = .02, HR = 20.59; Type 2 T helper cell: *P* = .01, HR = 24.83).

### 3.3 Inflammation and tumour immune features

**Figure 3:**
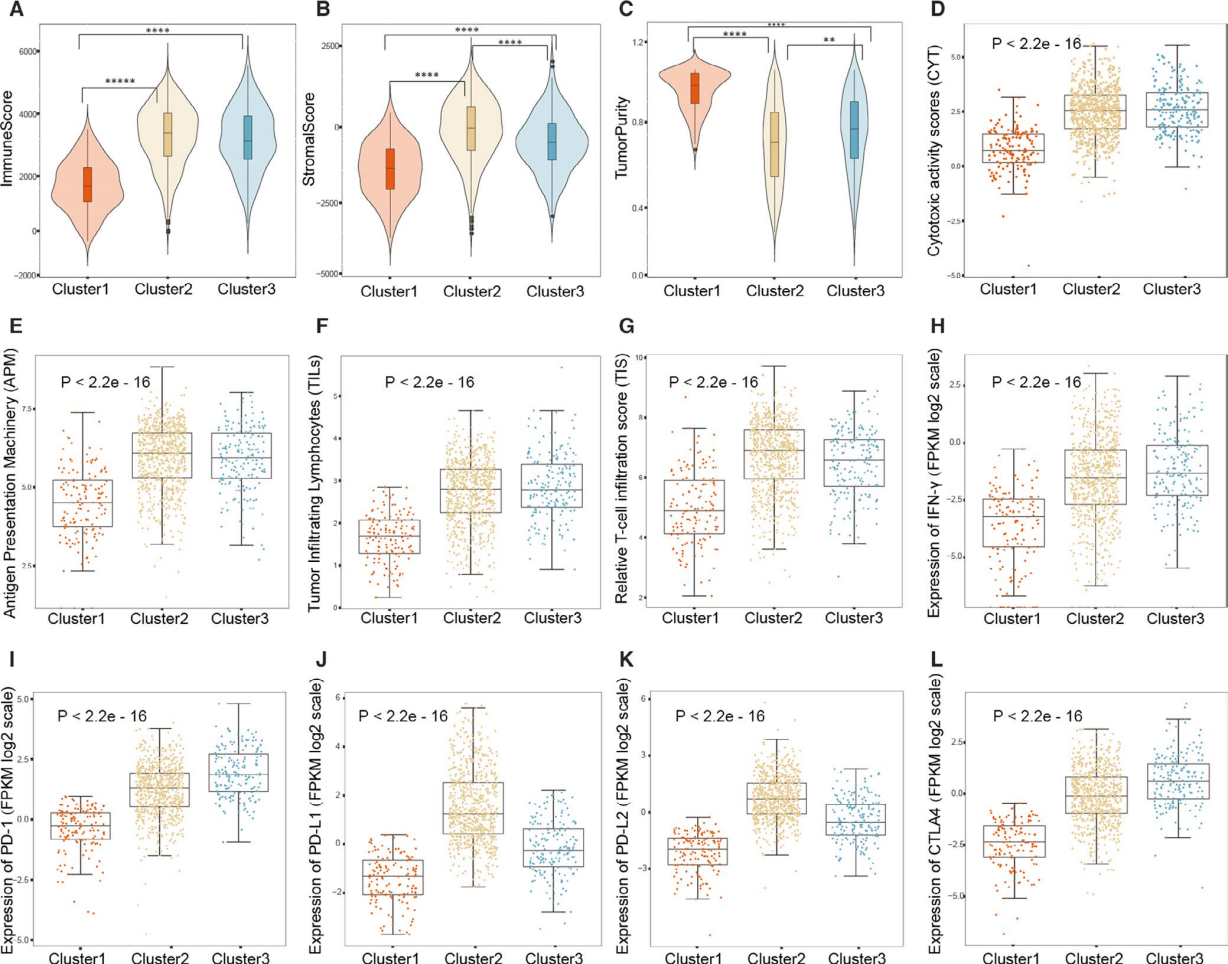
Inflammation and tumour immune features. A‐C, The ESTIMATE model was used to determine the stromal, immune scores and tumour purity of the immune clusters. D, Comparison of relative cytotoxic activity scores (CYT) between immune clusters. E, Relative antigen presentation machinery (APM) among immune clusters. F, Pathological evaluation of the percentage of tumour‐infiltrating lymphocytes (TILs) among immune clusters. G, Relative T cell infiltration score (TIS) among immune clusters. H, Expression of IFN‐γ between immune clusters. I‐L, immune checkpoint PD‐1, PD‐L1, PD‐L2 and CTLA4 expressions among immune clusters

To assess the abundance of immune and stromal cell infiltration, ESTIMATE was used. The stromal and immune cell scores were highest in cluster 2 followed by clusters 3 and cluster 1 respectively (Figure 3A,B). However, cluster 1 showed the highest tumour purity (Figure 3C). We further evaluated whether there was a correlation between the function of cytotoxic activity and higher levels of infiltration of immune cells. Compared with cluster 1, the outcome indicated higher cytotoxic potential in clusters 2 and 3, and there was barely any difference between cluster 2 and cluster 3 (*P* < 2.2e − 16) (Figure 3D). An antigen‐specific immune response was produced through effector CD8+ T cells, which were activated by MHC molecules presenting neoantigens or native intracellular proteins.

**Figure 4:**
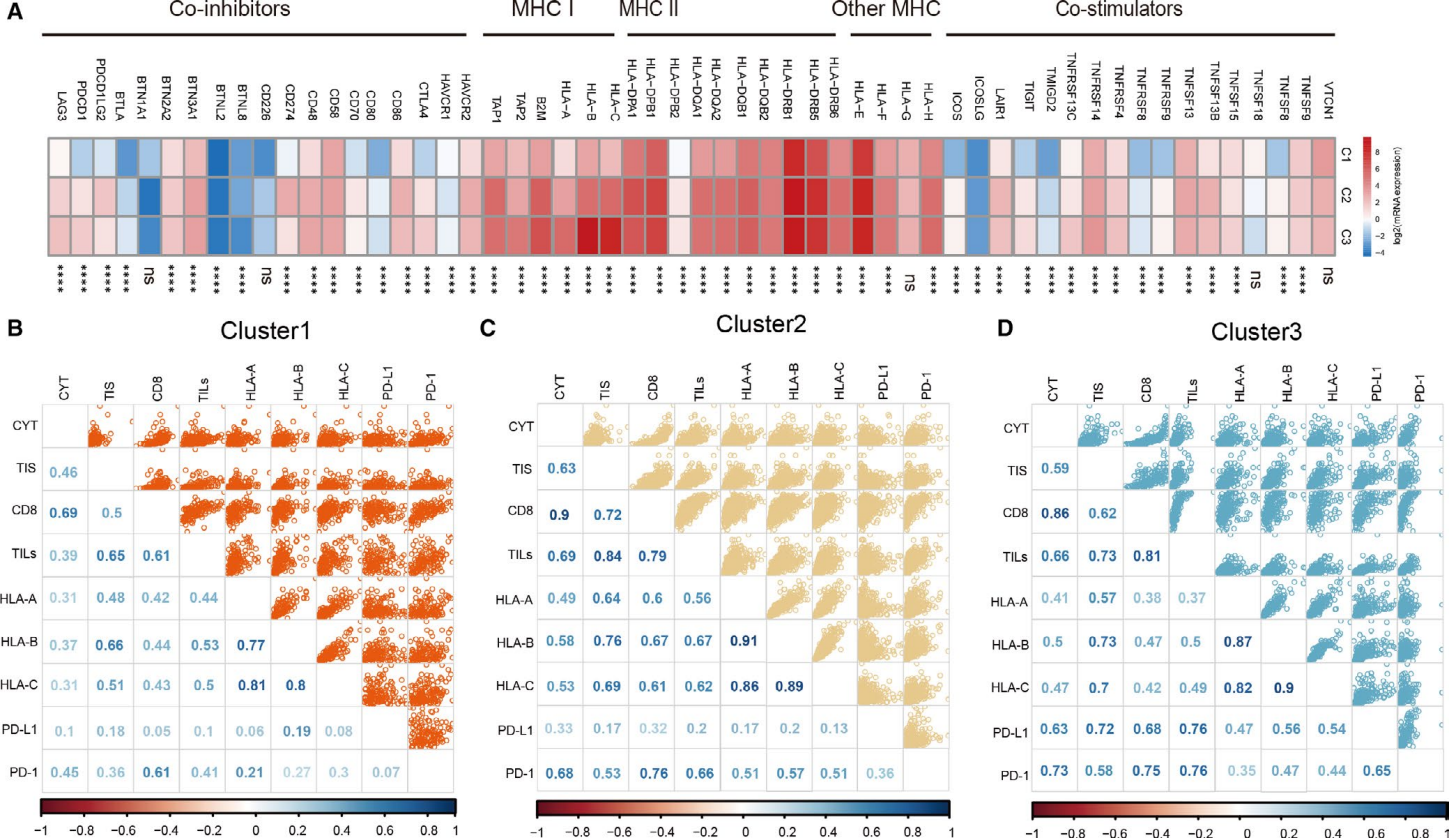
Potential extrinsic immune mechanisms. A, MHC molecules, immune co‐inhibitors and co‐stimulatory expression trends based on immune clusters. B‐D, Correlation matrix of local immune features and MHC‐I molecules across the immune cluster, as estimated by Pearson's correlation coefficients. CD8: ssGSEA result of activated CD8 T cell

The highest APM (*P* < 2.2e‐16) was in cluster 3 (Figure 3E). Higher TILs and TIS (*P* < 2.2e‐16) (Figure 3F,G), as well as INF‐γ^21^ were found in cluster 2 and cluster 3. Immune checkpoint blockade therapy through modulation of the PD‐L1 or PD‐1 axis has been shown to yield satisfactory patient outcome.^31^ We further quantified the expressions (PD‐1, PD‐L1, CTLA4, PD‐L2) of these key immune molecules. Among these, four checkpoints were markedly declined in cluster 1 (Figure 3I‐L).To compare the prevalence of these immune molecules, a data set of costimulatory and coinhibitory molecules was scrutinized.^32^ Cluster 1 had the lowest levels of costimulation (most *P* < .05) as shown in Figure 4A. Furthermore, a strong correlation was revealed between TILs scores and the proportion of CD8 T cells (ssGSEA CD8 score) in cluster 3 (Figure 4B‐D). Those in cluster 3 had the highest correlation (Pearson's correlation coefficients = .81), indicating possible organized processes and biological activity.

### 3.4 Functional analysis between immune microenvironments

**Figure 5:**
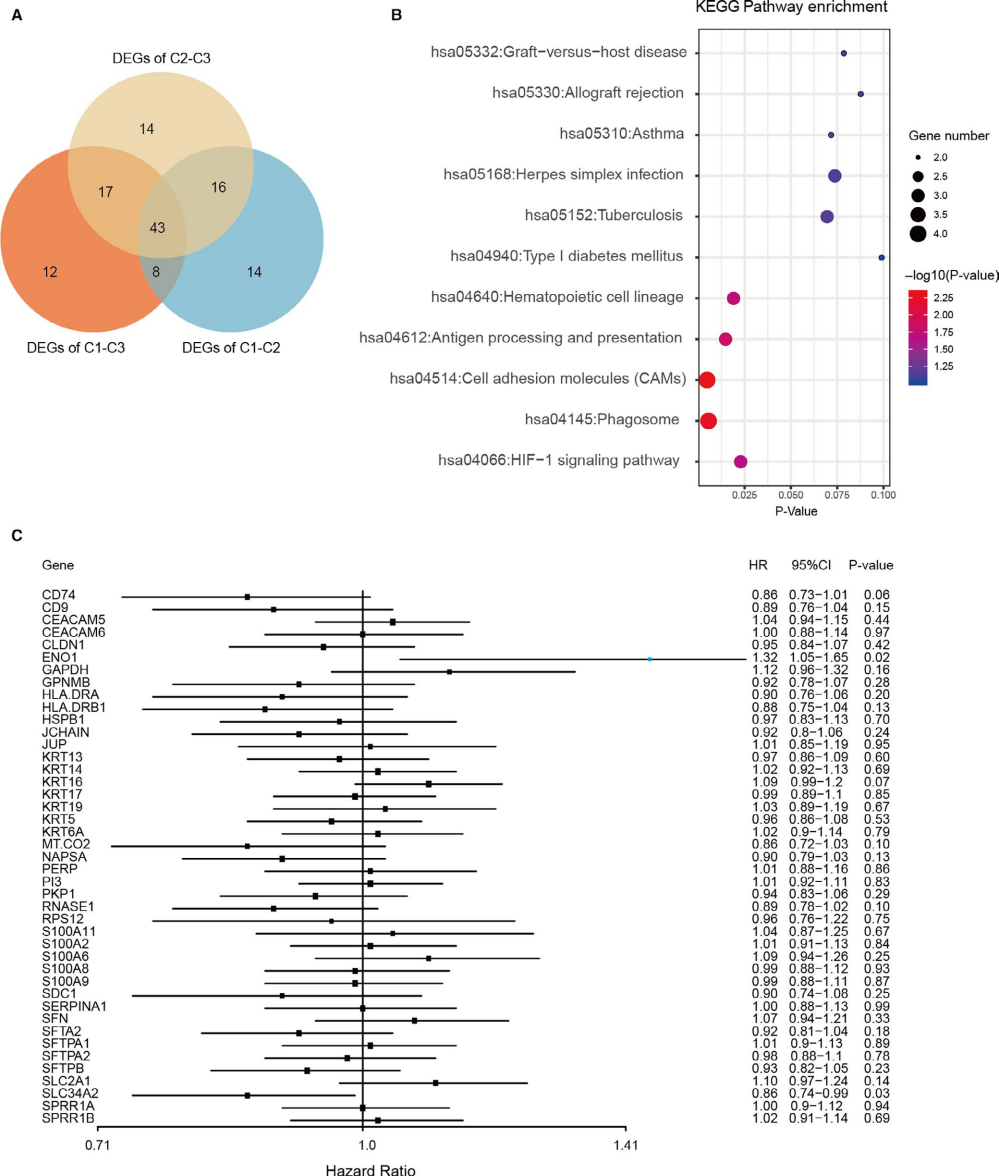
KEGG analysis of DEGs. A, Venn diagram illustrating the number of DEGs among the three immune clusters. B, KEGG pathway analysis. C, The forest plot for the DEGs. The univariable Cox model was used to determine statistical significance

Prognostic immune‐related genes were obtained by comparing three clusters. There were 43 overlapping DEGs obtained (Figure 5A). DAVID tool was used to implement a pathway enrichment analysis of DEGs (Figure 5B). The enrichment results showed the primary involvement of DEGs in cell adhesion molecules, HIF‐1 signalling pathway, as well as antigen processing and presentation. In addition, we established a univariate Cox regression model for the DEGs (Figure 5C), and ENO1 (HR = 1.32, *P* = .02, coefficient = 0.2771) and SLC34A2 (HR = 0.86, *P* = .03, coefficient = −0.1551) (Table S2) were considered as potential prognostic immune‐related genes.

### 3.5 Validation in the GEO data set

**Figure 6:**
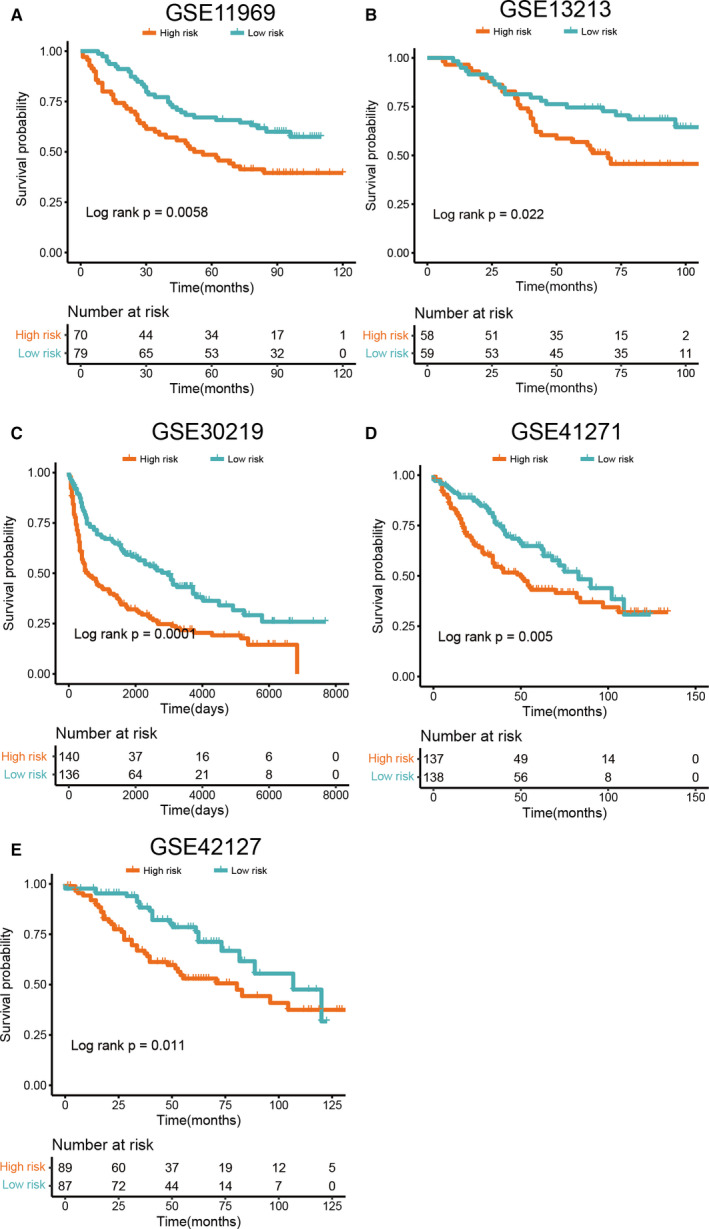
The Kaplan‐Meier survival analysis of the signature for both training set and testing sets. A, GSE11969 cohort, the number of patients in the High‐RS and Low‐RS subtypes are n = 82 and n = 81, respectively. B, GSE13213 cohort, the number of patients in the High‐RS and Low‐RS subtypes are n = 58 and n = 59, respectively. C, GSE30219 cohort, the number of patients in the High‐RS and Low‐RS subtypes are n = 154 and n = 153, respectively. D, GSE41271 cohort, the number of patients in the High‐RS and Low‐RS subtypes are n = 137 and n = 138, respectively. E, GSE42127 cohort, the number of patients in the High‐RS and Low‐RS subtypes are n = 89 and n = 87, respectively

To validate the prognostic importance of the two aforementioned gene, additional NSCLC data sets (accession numbers GSE11969, GSE13213, GSE30219, GSE41271 and GSE42127) were explored. The RS was calculated for all individuals in the validation data set (see Section 2). Based on the median RS value, the patients were grouped into high‐ and low‐risk categories. In all these classes, the survival outcome was significantly different (Figure 6A‐E). Unlike the high‐risk community, patients in the low‐risk group had significantly longer OS (GES11969: Log‐rank *P* = .0058; GSE13213: Log‐rank *P* = .022; GSE30219: Log‐rank *P* = .0001; GSE41271: Log‐rank *P* = .005; GSE42127: Log‐rank *P* = .011), indicating that ENO1 and SLC34A2 may be important in validation sets.

## 4 DISCUSSION

With the breakthroughs in human cancer immunotherapy, several immunotherapies have recently been clinically approved to treat multiple cancers.^33^ Unlike before, researchers have now focused more attention on the function of immune cell infiltration in the development, progression and prognosis of NSCLC.^34^ The heterogeneity of infiltrating subpopulations in NSCLC has been examined herein. Three immuno‐clusters were identified namely; cluster 1 (low infiltration), cluster 2 (heterogeneously infiltration) and cluster 3 (high infiltration). In addition, distinct immune subpopulations showed major variations in patient prognosis. This research identified two genes that were independently capable of predicting the survival of patients through integrative analysis of the TCGA cohort. A total of 1016 NSCLC microenvironments were divided into three heterogeneous clusters in our study (Figure 1A). The lowest degree of immune cell infiltration was in cluster 1, whereas the highest was observed in cluster 3. In estimating the relative proportions of immune cell populations, the CIBERSORT approach was used (Figure 1B). The most abundant immune cells were the macrophage population, including M0 and M2 macrophages.^35^ Notably, the highest relative abundance of CD8 T cells was observed in cluster 3 (Figure 1B). Three immune clusters and tumour subtypes showed significant differences in patients’ OS (Figure 2A,B). Cluster 1 was significantly associated with a poorer prognosis, whereas cluster 3 was associated with a more favourable prognosis (*P* = .003).

Those in cluster 3 also had higher levels of CD8 T cells infiltration and immune checkpoints (Figures 1A and 3I‐L).^36^ Given that higher levels of checkpoint molecules were present in clusters 2 and 3, we infer that effector T cells are responsible in a quantity‐dependent manner for checkpoint stimulation.^37^ Besides, significantly higher rates of survival with high‐immune infiltration level were observed in cluster 3 cohort (Figure 2A,B). We subsequently assessed the prognostic value of the immune cell profiles and checkpoint molecules and found that 2 immune cells exhibited significant prognostic values (Type 2 T helper cell: HR = 24.83, *P* = .01; Gamma delta T cell: HR = 20.59, *P* = .02) (Figure 2C). Several studies show that Type 2 T helper cells can cause pulmonary fibrosis‐aggravating immune responses.^38^, ^39^

Subsequently, the distribution of stromal, immune and tumour purity levels was studied. Cluster 1 had a significantly lower immune and stromal score (Figure 3A,B), but with the highest purity of the tumour (Figure 3C). These observations confirm that the highest tumour cell aggregation in cluster 1 is associated with poor survival. A possible predictor for the assessment of the immune microenvironment may be a cytotoxic activity, which indirectly represents the degree of oedema around the tumour. Our results show that clusters 2 and 3 have higher CYT levels (Figure 3D), which is consistent with previous studies that showed an association between high CYT levels and higher patient OS.^40^ The MHC is a vital effector of the immune response as an antigen‐presenting molecule.^41^, ^42^ Cluster 1 showed the lowest APM levels in our sample (Figure 3E). TILs, TIS and IFN‐γ were also present in cluster 1 in low levels but were higher in clusters 2 and 3, respectively (Figure 3F‐H). Subsequent studies have shown that by enhancing the activation of the MHC‐I antigen processing and presentation pathway, IFN‐γ can facilitate tumour removal. These results are consistent with the poor survival of cluster 1, often characterized by lower degrees of infiltration by immune cells, as well as lower levels of immune checkpoints and immune cell infiltration (Figure 3I‐L). In 2004, Gavin P. Dunn et. al reported that tumour progression or clearance appeared to be dependent on immune system‐tumour interactions.^43^ Using cancer immunoediting, they divided the tumours into three types including elimination, equilibrium and escape.^43^ The elimination and equilibrium tumours represent tumours with complete immunoediting process or dynamic equilibrium, respectively.

However, escape type tumour cells develop a myriad of immunoevasive strategies that result in unchecked tumour proliferation. These mechanisms involve aberrant tumour recognition by immune effector cells (such as loss of MHC components and subsequent antigen expression), and the development of IFN‐γ insensitivity.^44^, ^45^ This is consistent with the findings in Figure 4A. In cluster 1, MHC‐I was down‐regulated, suggesting that it could be an escape tumour type. In the three clusters, we further determined the association between immune infiltration factors and observed visible differences. Compared with the more positive associations in clusters 2 and 3, possible disrupted mechanisms and biological signalling pathways were implied by the relatively weak ones in cluster 1.

We calculated three classes of overlapping differential genes (Figure 5A). And the 43 DEGs were used to a complete KEGG pathway analysis (Figure 5B). The results showed that these genes are enriched in several important pathways, such as antigen processing and presentation pathway. Cox analysis was then used to classify the prognostic immune‐related genes. Two genes were closely linked to patient outcomes among the DEGs (ENO1: HR = 1.32, *P* = .02; SLC34A2: HR = 0.86, *P* = .03) (Figure 5C). In several types of cancer, including cholangiocarcinoma, breast cancer, head and neck cancer, leukaemia, lung cancer, pancreatic cancer and melanoma, ENO1 has shown diagnostic and prognostic importance.^46^ Previous studies in NSCLC have shown that ENO1 is a crucial oncogenic molecule significantly associated with tumorigenesis and metastasis by promoting neoplastic transformation.^47^, ^48^, ^49^ Interestingly, its ability to trigger a strong cellular immune response makes it a potential immunotherapy target.^46^, ^50^ SLC34A2 is a membrane protein that, through sodium ion co‐transport, mediates the transport of inorganic phosphate into epithelial cells and also plays a suppressive role in NSCLC tumorigenesis.^51^, ^52^ These studies support our findings that ENO1 and SLC34A2 are possible NSCLC markers. Finally, we developed a RS model for survival prediction through the integration of gene expression and NSCLC clinical profiles. In independent verification sets, the RS signature could precisely predict the OS of individuals. In conclusion, by analysing a total of 2056 NSCLC samples, we identified three immune clusters and prognostic immune signatures. This research gives greater insights into NSCLC's fundamental molecular mechanisms. The immune cell profiling can clarify the immunophenotype of NSCLC, provide prognostic information and predict the efficacy of immunotherapy.

## CONFLICTS OF INTEREST

The authors declare no conflicts of interest.

## AUTHOR CONTRIBUTION

**Kai Mi:** Data curation (lead); Methodology (lead); Software (lead); Writing‐original draft (lead). **Fuhui chen:** Conceptualization (supporting); Investigation (lead); Supervision (equal); Writing‐original draft (supporting); Writing‐review & editing (supporting). **Zhipeng Qian:** Data curation (supporting); Investigation (supporting); Methodology (supporting); Software (supporting). **Jing Chen:** Data curation (supporting); Methodology (supporting); Project administration (supporting); Validation (supporting); Writing‐review & editing (supporting). **Dongxu Lv:** Data curation (supporting); Investigation (supporting); Methodology (supporting). **Chunlong Zhang:** Supervision (supporting); Writing‐review & editing (supporting). **Yanjun Xu:** Supervision (supporting); Validation (supporting). **Hongguang Wang:** Software (supporting). **Yuepeng Zhang:** Data curation (supporting). **Yanan Jiang:** Conceptualization (supporting); Writing‐review & editing (lead). **Desi Shang:** Conceptualization (lead); Writing‐original draft (lead); Writing‐review & editing (lead).

## Supporting information

Fig S1

Table S1

Table S2

## Data Availability

The data that support the findings of this study are available from the corresponding author upon reasonable request.
