# Supplementary material for: Characterizing heterogeneity of non‐small cell lung tumour microenvironment to identify signature prognostic genes
Source: J Cell Mol Med. 2020 Nov 12;24(24):14608–18. doi: 10.1111/jcmm.16092 (PMC7754023; doi:10.1111/jcmm.16092)
Supplement: Supplementary file 1 — Fig S1 [file JCMM-24-14608-s001.pdf]

Univariate and multivariate cox proportional hazards model for OS

Variables		Univariate		Multivariate	
		HR(95% CI)	P-value	HR(95% CI)	P-value
Age(years)		1.17(0.92 - 1.51)	0.006	1.32(0.82 - 2.11)	0.25
Stage		2.99(0.67- 13.40)	2e-12	0.51(0.06 - 4.56)	0.54
Gender		1.15(0.93 - 1.41)	0.2	Not included	
microenvironment phenotype	Cluster 1	Ref		Ref	
	Cluster 2	0.79(0.60 - 1.04)	0.1	Not included	
	Cluster 3	0.58(0.40- 0.83)	0.003	0.66(0.46 - 0.96)	0.03
